# Concordance between immunohistochemistry and MSI analysis for detection of MMR/MSI status in colorectal cancer patients

**DOI:** 10.1186/s13000-024-01571-5

**Published:** 2024-11-29

**Authors:** Muhammad Ishaque Faizee, NorLelawati A. Talib, Asmah Hanim Bt. Hamdan, Nor Zamzila Bt. Abdullah, Bilal Ahmad Rahimi, Ahmed Maseh Haidary, Ramin Saadaat, Ahmed Nasir Hanifi

**Affiliations:** 1 Department of Pathology, Faculty of Medicine, Kandahar University, Kandahar, Afghanistan; 2 Department of Pathology and Laboratory, Kulliyyah of Medicine, International Islamic University Malaysia (IIUM), Kuantan, Pahang Malaysia; 3 Department of Pediatrics, Faculty of Medicine, Kandahar University, Kandahar, Afghanistan; 4 Department of Pathology and Clinical Laboratory, French Medical Institute for Mother and Children (FMIC), Kabul, Afghanistan; 5 Central Public Health Laboratory, Ministry of Public Health, Kabul, Afghanistan

**Keywords:** MMR, MSI, IHC, MSI analysis, Colorectal cancer

## Abstract

**Background:**

Recently, screening of colorectal cancer (CRC) patients for mismatch repair/microsatellite instability (MMR/MSI) status is widely practiced due to its potential predictive and prognostic roles and a screening tool to reveal Lynch Syndrome (LS). The purpose of the study was to evaluate concordance between immunohistochemistry (IHC) and MSI analysis methods for detection of MMR/MSI status in colorectal cancer patients in Kuantan, Pahang.

**Methods:**

Fifty selected CRC cases of deficient mismatch repair (dMMR) and proficient mismatch repair (pMMR) which were identified immunohistochemically in the previous study were subjected to MSI analysis. MSI Analysis System 1.2 (Promega) was utilized.

**Results:**

The results of MSI analysis method showed MSI-High: 26% (13/50), MSI-Low: 6% (3/50), and Microsatellite Stable: 68% (34/50). The concordance was perfect (0.896, Kappa value) between MSI analysis and IHC methods for the assessment of MMR/MSI status in CRC patients. The discordance was only 4% (2/50). MSI analysis identified all dMMR cases determined by IHC except one case. The obtained frequency of dMMR and pMMR patients was 11.4% (14/123) and 88.6% (109/123) by IHC method, respectively.

**Conclusion:**

Our findings support the universal practice of evaluating the MMR/MSI status in all newly diagnosed CRC patients. Based on the perfect concordance of two methods, the method of choice is based on the availability of expertise and equipments. IHC is highly appreciable method due to its feasibility and reproducibility.

## Introduction

There might be well established distinctions in the genetic landscape, the response to immunotherapy and chemotherapy, as well as the staging and classification of tumors based on the MMR/MSI status. The identification of MMR/MSI status has diagnostic, therapeutic and prognostic importance in colorectal cancer and other cancers [1]. Early-stage dMMR/MSI-H tumors are reported to show better prognosis [2] and be less responsive to fluorouracil compared to MSI-L/MSS tumors. It has significant role in the pathogenesis of LS; characterized by germline mutations in any one or more of the MMR genes (MLH1, MSH2, MSH6, and PMS2 and rarely, PMS1) [3].

Sporadic CRCs, in contrast to LS, are mainly due to somatic hyper methylation of the MLH1 promoter and linked with somatic BRAF mutation [4–6]. Therefore, assessment of all newly diagnosed CRC with MSI analysis and/or IHC is largely recommended [7–10]. However, there is a lack of international consensus on the use of methods to be applied concurrently or sequentially for the assessment of MMR/MSI status in CRC patients.

IHC, applied on paraffin-embedded tissue specimen, indirectly specifies the possible gene responsible for the MMR deficiency [11, 12] through an assessment of the corresponding protein products [13]. MSI analysis, applied on extracted tumour DNA, detects MSI status by Polymerase Chain Reaction (PCR) amplification of microsatellite areas, in which the length of microsatellite areas in tumour cells and normal cells from nearby normal tissue are compared [14, 15]. Molecularly, various combinations of mononucleotide and dinucleotide markers or mononucleotide markers have been suggested for the assessment of MSI status in CRC patients [16–19].

MMR/MSI detection either with MSI analysis or IHC have both advantages and disadvantages [12, 20]. During IHC reporting, certain pitfalls such as heterogeneous staining, zonal decreased internal control tissue staining, cancer cells exhibiting strong nuclear staining with adjacent weak/absent staining, and intratumoral lymphocytes are the potential concerns linked with variable IHC reporting [21]. However, heterogeneous staining (zonal and/or focal found in the same cancerous tissue) in IHC is not believed to be always artifactual [22]. Moreover, misdiagnosis of dMMR/MSI-H status is another concern which is reported in metastatic CRC patients [23].

Since the results of MMR protein and MSI analysis have considerable impacts on the treatment decisions of CRC patients in clinical setting, it is essential to identify the applicable and reproducible method in our local setting. To our knowledge, there is no national consensus on the choice and accepted method that is applicable for Malaysian CRC patients. In this study, we report the concordance between IHC and MSI analysis for MMR/MSI status in CRC patients.

## Materials and methods

### Study design and sample collection

This study was a cross-sectional study on CRC cases. All 123 CRC cases diagnosed in Hospital Tengku Ampuan Afzan (HTAA) and International Islamic University Malaysia Medical Center (IIUMMC) between 1st January 2017 to 31st December 2018 were identified through the pathology records of the patients. For each case, three selected FFPE blocks were retrieved.

### Immunohistochemistry for mismatch repair proteins

IHC was performed to assess the dMMR status of the CRC cases. Immunohistochemical staining was done manually utilizing a polymer detection kit (Envision FLEX, DAKO, Denmark) with the following reagents: wash buffer (20X), antigen retrieval solution (50X), peroxidase-blocking reagent, mouse (linker) for MLH1, HRP (Horseradish peroxidase) and 3.3’-diaminobenzidine tetrahydrochloride chromogen. The details of the procedure are mentioned previously [24].

The cases that were reported as dMMR (abnormal) on preliminary assessment were re- assessed. The MMR protein was reported as normal (pMMR), and abnormal (dMMR). The stromal and epithelial cells of the colonic mucosa were considered as the internal control.

### MSI analysis

MSI analysis was performed by the PCR capillary electrophoresis (PCR-CE) method utilizing MSI Analysis System Version 1.2 (Promega), which comprised seven markers including five mononucleotide repeat markers (BAT-25, BAT-26, NR-21, NR-24, and MONO-27) and two pentanucleotide repeat markers (Penta C and Penta D) [25]. Of the 123 CRC cases, MSI analysis was carried out for 50 selected CRC cases. These CRC cases consist of 13 dMMR and 37 pMMR cases as identified by the earlier IHC analysis. MSI analysis was performed on 50 cases (we included all 13 dMMR cases and randomly selected 37 pMMR cases). For MSI analysis, all these 50 cases were represented with normal and tumour tissues except one which was represented by only tumour tissue.

Genomic DNA (gDNA) was extracted from 5 μm sections of FFPE tissue with the Maxwell 16 instrument (Promega, Southampton, UK) using the Maxwell^®^ RSC DNA FFPE Kit. DNA was quantified using Quantus™ Fluorometer. The following optimized protocol of Master Mix for PCR was utilized which illustrates the component volumes per sample when using a DNA template volume of 2 µl (0.5ng/ µl) in a 6 µl reaction volume comprising of Nuclease-Free Water (2.9 µl), Gold STHR 10X Buffer (0.5 µl), MSI 10X Primer Pair Mix (0.5 µl), and Go Taq^®^ MD x Hot Start Polymerase 7.9/µl (0.1 µl).

After the completion of the thermal cycling protocol, the PCR tubes were submitted for capillary electrophoresis along with Internal Lane Standard 600 (ILS 600). The results were analysed using Genemarker software. MSI was defined as any marker with the highest peak shifted three or more base pairs (≥ 3 bp) when compared to the same marker in the normal sample. Instability in two or more of the five mononucleotide markers was classified as MSI-H, instability in one mononucleotide marker was classified as MSI-L and absence of instability in any of the markers was classified as MSS [19, 22, 26]. Overall, cases were not included in the study if all five markers were not interpretable, unless at least two of those that were interpretable could be analysed as showing instability, reflexing MSI-H status.

### Statistical analysis

Statistical Package for the Social Sciences (SPSS, version 23.0) was utilized for statistical analyses. Data were analysed using Pearson’s chi-square or Fisher exact test to assess the association of the categorical variables. Additionally, Cohen’s kappa (κ) coefficient statistic was used to observe the concordance between IHC and MSI analysis methods. The κ value was interpreted as: almost perfect agreement (0.81-1.00); substantial agreement (0.61–0.80); moderate agreement (0.41–0.60); fair agreement (0.21–0.40), slight agreement (0.01–0.20) and poor agreement (< 0). Statistical significance was set as *p* < 0.05 [26–28].

## Results

### Immunohistochemistry for mismatch repair proteins

**Fig. 1:**
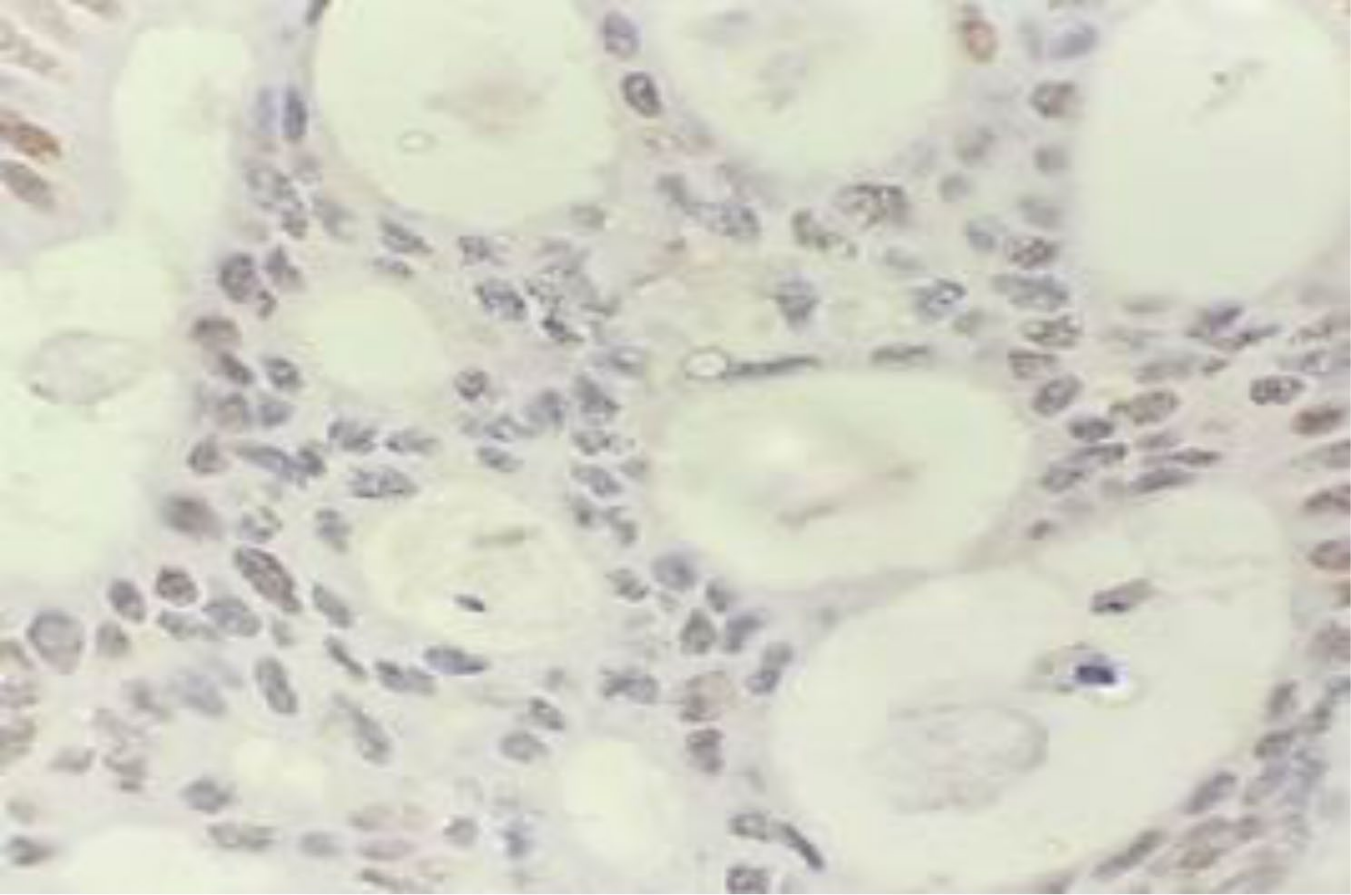
IHC staining for PMS2 protein re-classified from dMMR to pMMR due to heterogenous staining

**Fig. 2:**
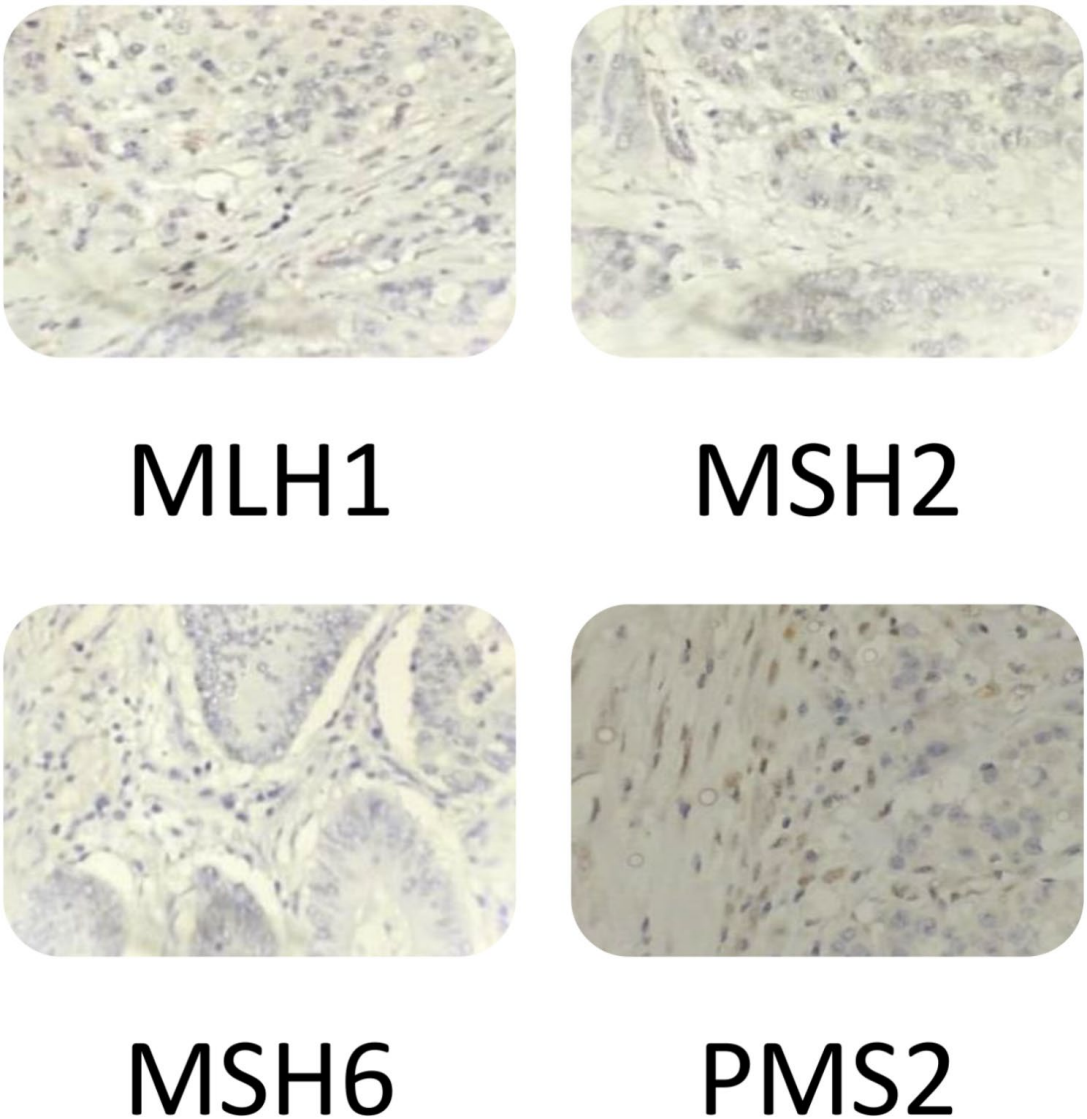
The loss of different MMR Proteins by IHC

Preliminary Immunohistochemical study revealed 17.07% (21 out of 123) cases that showed the loss of one or more MMR protein expression and were dMMR. During re-assessment of the slides, 7 cases in which previously reported as dMMR, were re-classified as pMMR (normal). Hence, dMMR cases were 11.4% (14 out of 123). The findings of the review of MMR protein IHC slides and their corresponding MSI results are shown in the following Table 1. Also, the below figure demonstates the example of MMR protein by IHC which was re-classified as pMMR due to heterogenous staining (Fig. 1). The loss of different MMR proteins by IHC are shown in Fig. 2.

**Table 1:** Findings of the review of MMR IHC slides and corresponding MSI results

No	Preliminary IHC	Loss of MMR protein by IHC	Reviewed IHC results	MSI status by PCR	Loss of MSI marker by PCR
1	dMMR	MSH2, PMS2	pMMR	MSI-low	BAT-25 loss
2	dMMR	PMS2	pMMR	MSI-low	NR-24 loss
3	dMMR	MSH2	pMMR	MSI-Stable	None
4	dMMR	MLH1	pMMR	MSI-Stable	None
5	dMMR	MSH2	pMMR	MSI-Stable	None
6	dMMR	MSH2	pMMR	MSI-Stable	None
7	dMMR	MSH2	pMMR	MSI-Stable	None

### MSI status

Using the PCR-CE method, the MSI status of the 50 selected CRC cases was obtained. The MSI analysis categorized 13/50 (26%) cases as MSI-H, 3/50 (6%) cases as MSI-L, and 34/50 (68%) cases as MSS tumours (Table 2).

**Table 2:** Results of microsatellite status in the study samples (*n* = 50)

MSI Analysis	Cases *n*(%)
High (MSI-H)	13 (26)
Low (MSI-L)	3 (6)
Stable (MSS)	34 (68)

MSI: Microsatellite instability; MSS; Microsatellite Stable; n: number of cases

### The electropherogram of the positive MSI markers

**Fig. 3:**
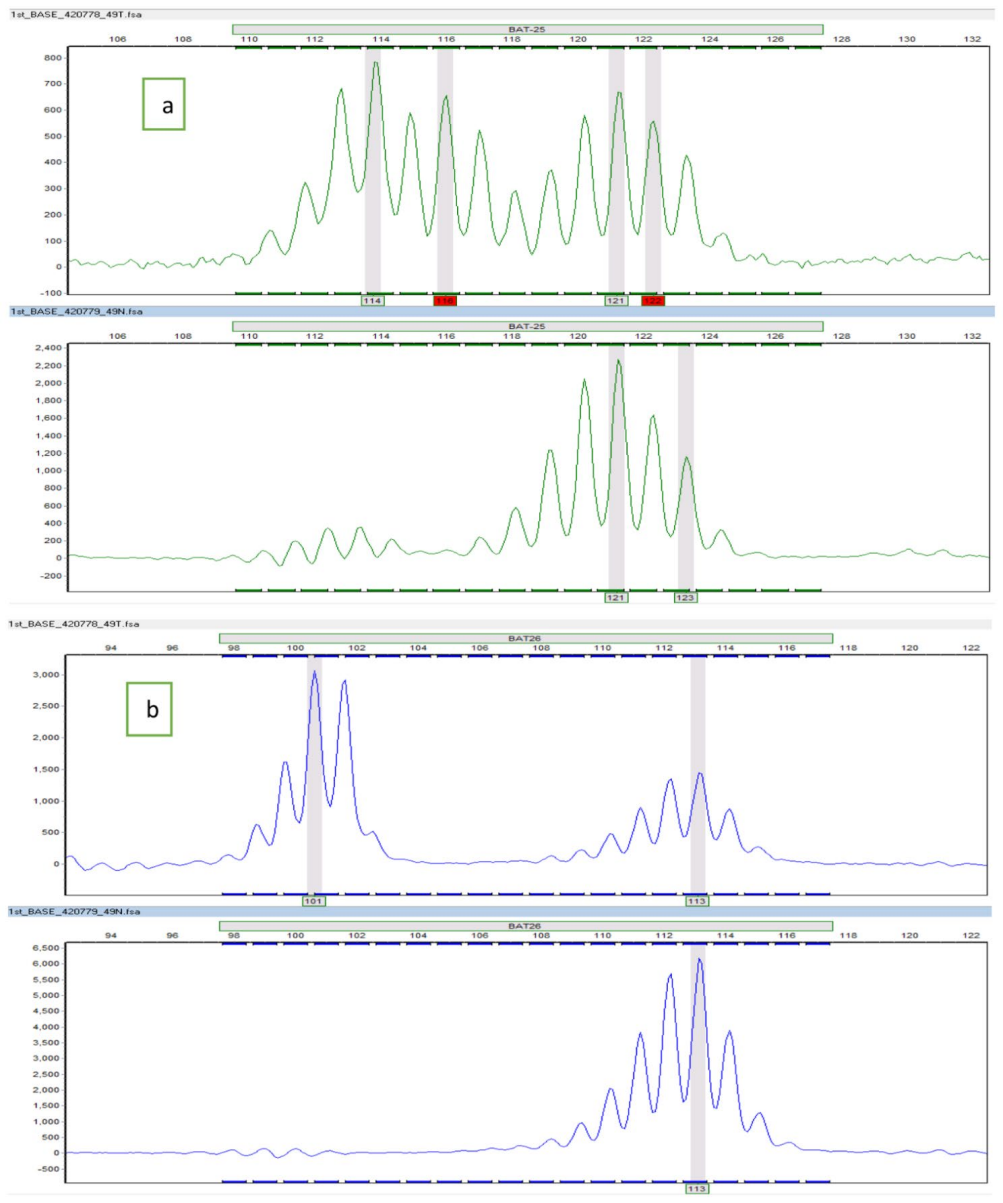
Representative electropherograms of the positive MSI markers; (**a**) represents normal (lower) and corresponding tumour (upper) tissues with their alleles (prominent peaks) at Bat-25 locus; (**b**) represents normal (lower) and corresponding tumour (upper) tissues with their alleles (prominent peaks) at Bat-26 locus. The tumour tissues exhibited extra alleles not present in normal tissues

**Fig. 4:**
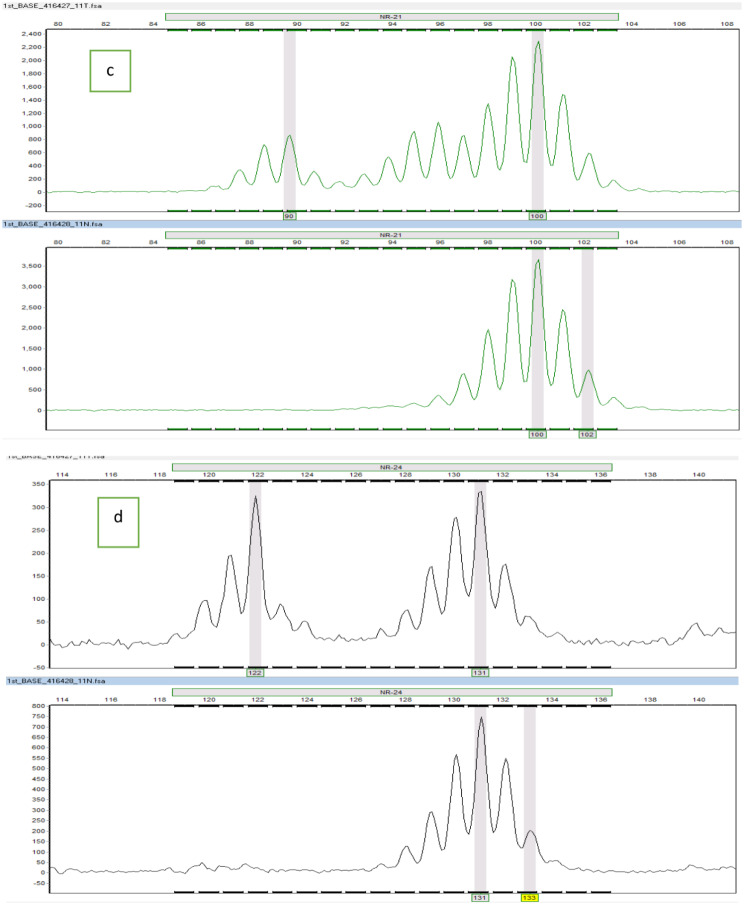
Representative electropherograms of the positive MSI markers; (**c**) represents normal (lower) and corresponding tumour (upper) tissues with their alleles (prominent peaks) at NR-21 locus; (**d**) represents normal (lower) and corresponding tumour (upper) tissues with their alleles prominent peaks) at NR-24 locus. The tumour tissues exhibited extra alleles not present in normal tissues

**Fig. 5:**
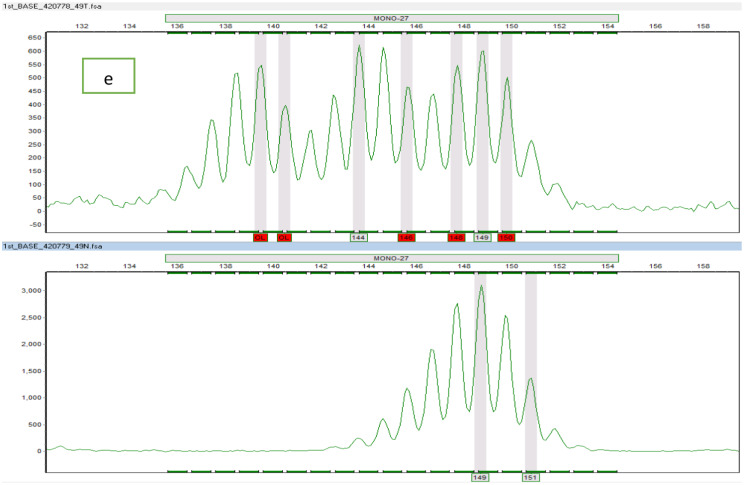
Representative electropherograms of the positive MSI markers; (**e**) represents normal (lower) and corresponding tumour (upper) tissues with their alleles (prominent peaks) at MONO-27 locus. The tumour tissue exhibited extra alleles not present in normal tissue

The representative electropherograms of the positive BAT-25, BAT-26, NR-21, NR-24, and MONO-27 markers are shown in Figs. 3 and 4, and 5.

### Profiles and distribution of the MSI positive markers

**Fig. 6:**
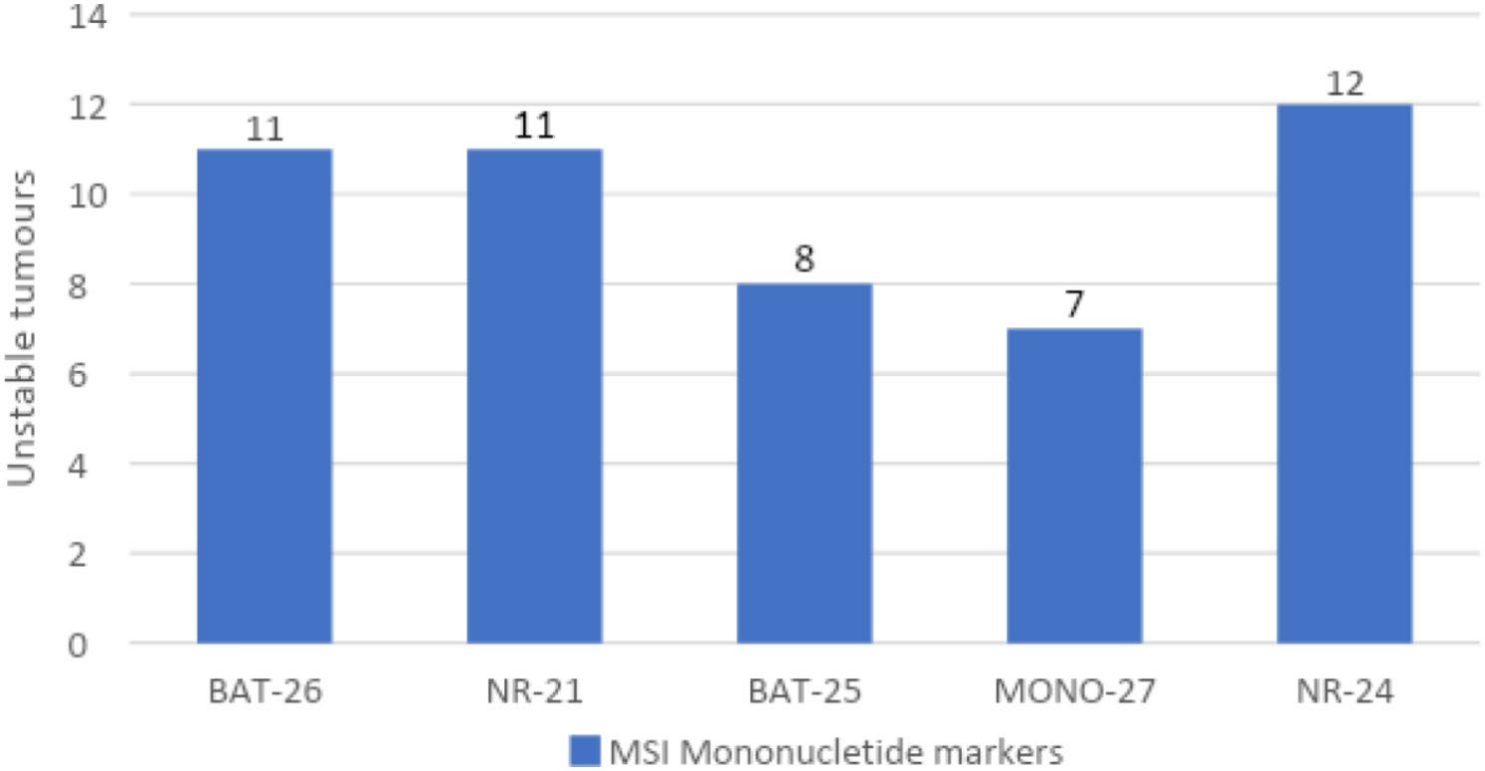
Individual distribution of unstable mononucleotide markers found in MSI positive CRC cases

**Fig. 7:**
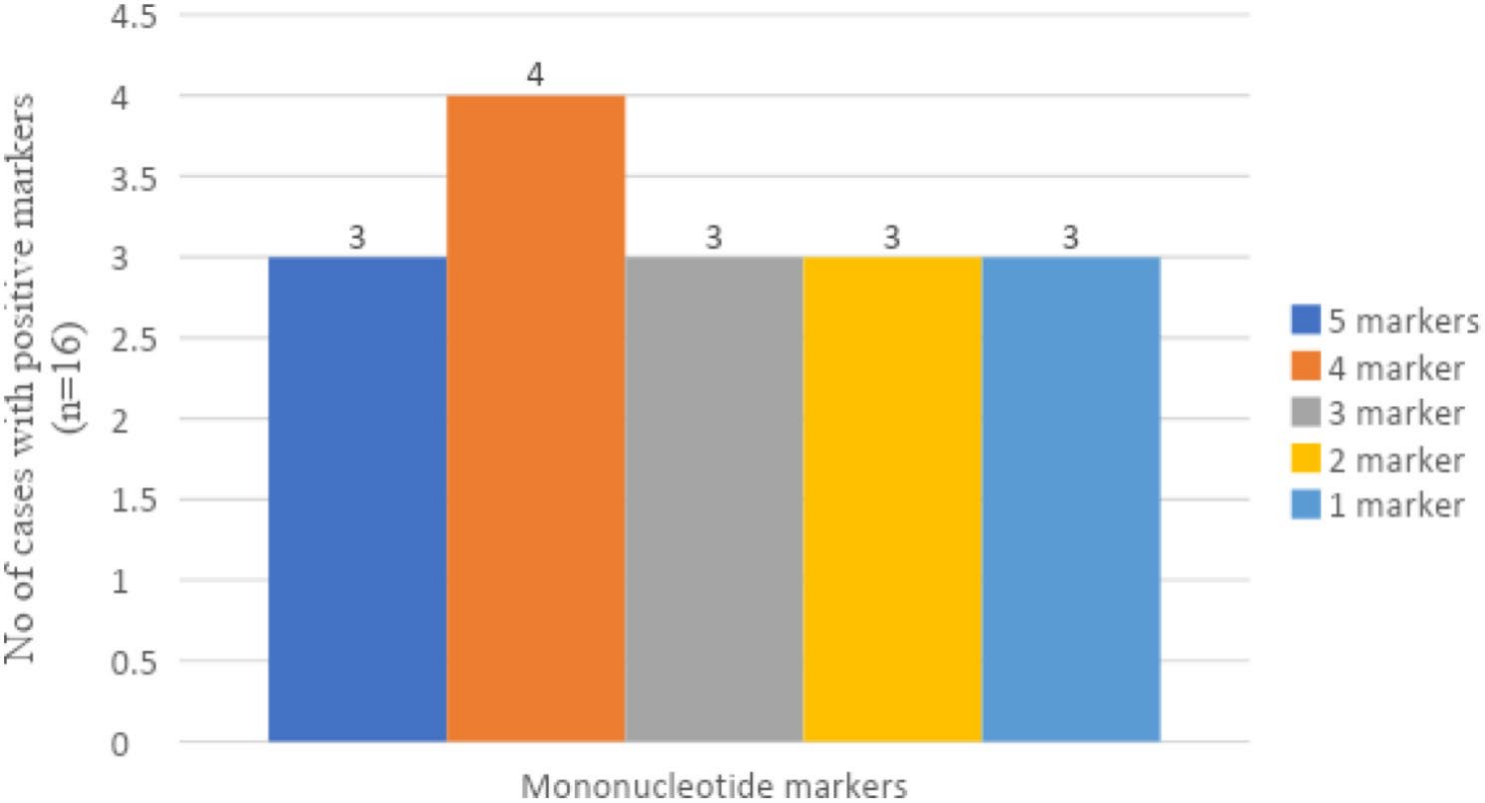
MSI positive markers grouping distribution. *n* = 3 showed five marker positivity (NR-24, Bat-25, Bat-26, MONO-27, and NR-21); there are 2, 2, and 3 different marker combinations in cases with 4, 3, and 2 markers positivity, respectively

The NR-24 was the most frequent unstable mononucleotide marker present in 12 of the 16 MSI positive (MSI-H and MSI-Low) CRC cases. Combined BAT-26 and NR-21 mononucleotide markers were unstable in 11 cases whilst BAT-25 and MONO-27 were unstable in 8 and 7 cases, respectively. Additionally, 3 cases expressed instability for all the five markers, 4 cases with 4 unstable markers, 3 cases with 3 unstable markers, 3 cases with 2 unstable markers, and 1 case with 1 unstable marker. The details of the individual unstable mononucleotide marker distribution are shown in Fig. 6 and the details of the MSI positive markers grouping distribution are shown in Fig. 7.

### Association of the clinicopathological features of CRC cases with the MSI status

We studied the patient’s demographic and association between clinicopathological features and MSI status in CRC. There was no statistically significant association between clinicopathological characteristics of the CRC cases and their MSI status categorized as MSS/MSI-L or MSI-H. The detailed result is shown in the Table 3.

**Table 3:** Association between clinicopathological features and MSI status in CRC

Variables	Total(*n* = 50) *n* (%)	MSS/MSI-L(*n* = 37) *n* (%)	MSI-H(*n* = 13) *n* (%)	*p*-value
Mean age (yrs., range)	62.18(31–86)			
Age(yrs.) ≤ 50 > 50	7(14) 43(86)	4(10.8) 33(89.2)	3(23.1) 10(76.9)	0.357
Gender Female Male	16(32) 34(68)	10(27) 27(73)	6(46.15) 7(53.85)	0.301
Race Malay Chinese	40(80) 10(20)	29(78.4) 8(21.6)	11(84.6) 2(15.4)	0.629
Site of tumour Right-sided Left-sided	13(26) 37(74)	7(18.9) 30(81.1)	6(46.15) 7(53.85)	0.073
pTNM staging I + II III + IV	26(52) 24(48)	19(51.35) 18(48.65)	7(53.85) 6(46.15)	0.877
Bowel wall invasion pT1 + pT2 pT3 + pT4	11(22) 39(78)	10(27.03) 27(72.97)	1(7.7) 12(92.3)	0.248
Lymph node metastasis pN- pN+	27(54) 23(46)	20(54.1) 17(45.9)	7(53.85) 6(46.15)	0.990
Lymphovascular Invasion NO YES	38(76) 12(24)	29(78.4) 8(21.6)	9(69.2) 4(30.8)	0.707
Histological Differentiation Well + Moderate Poor	48(96) 2(4)	36(97.3) 1(2.7)	12(92.3) 1(7.7)	0.456
Histological Type Mucinous Non-mucinous	5(10) 45(90)	3(8.1) 34(91.9)	2(15.4) 11(84.6)	0.595

pN-/+: pathological Node negative/positive; pT: pathological Tumour; pTNM: pathological Tumour Node Metastasis; MSS-L: Microsatellite Stable Low; MSI-H: Microsatellite High; n: number of cases; p is significant at value ˂0.05

### Distribution of MSI status and its corresponding MMR proteins of the MSI positive cases

**Fig. 8:**
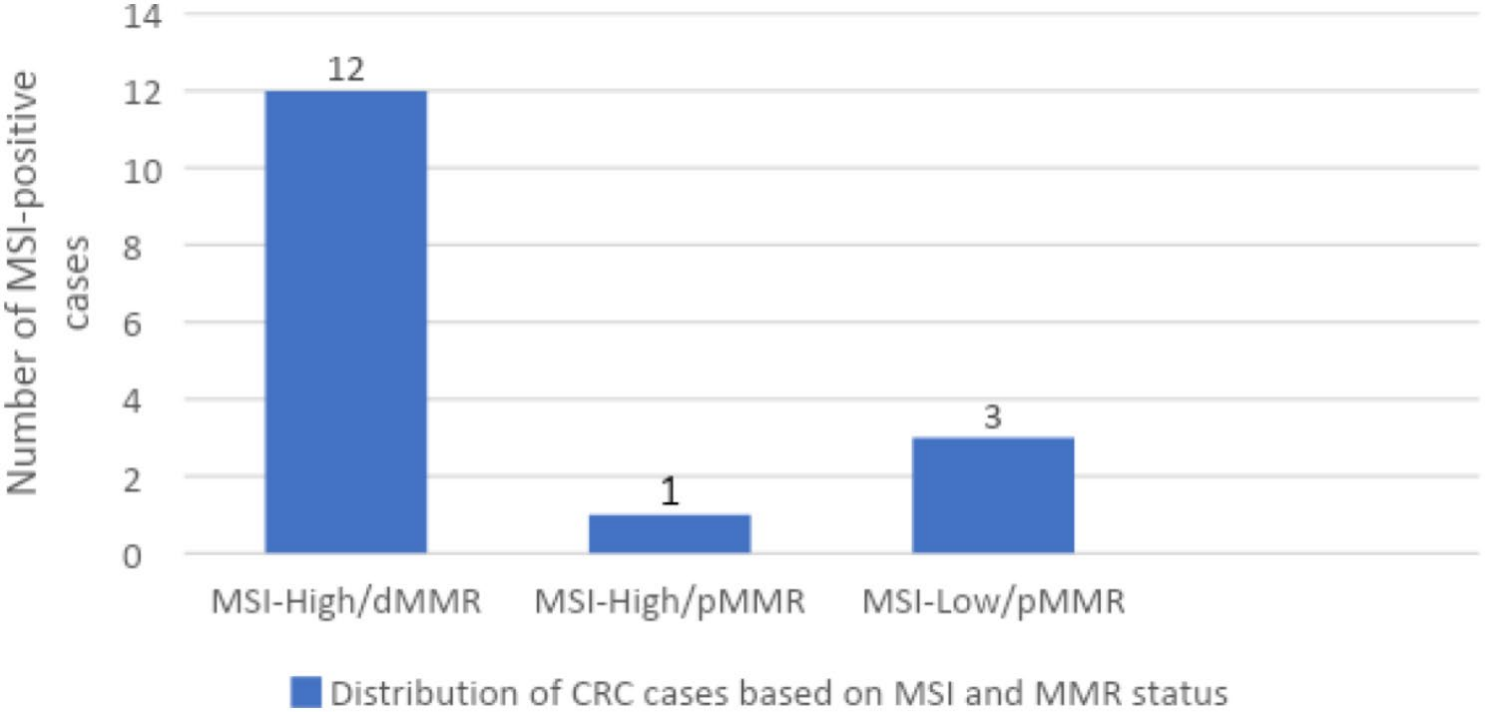
MSI-positive cases with MSI-H/MSI-L and pMMR/dMMR status

Of these 16 cases of MSI positive (either MSI-H or MSI-low), there was one case of MSI-H but pMMR status. Alternatively, all the 3 MSI-L cases were pMMR by IHC. The distribution of all cases labeled as per their MSI and MMR status are displayed in Fig. 8; Table 4.

**Table 4:** MSI positive cases with corresponding MMR proteins result

Patient No	Detection results of MSI		IHC results of MMR proteins	
	Unstable MSI markers	MSI status	Loss of MMR proteins by IHC	MMR protein Status
1	BAT-26, NR-21, MONO-27, NR-24	High	MLH1/PMS2	dMMR
2	BAT-26, NR-21, BAT-25, NR-24	High	MLH1/PMS2	dMMR
3	BAT-26, NR-21, MONO-27	High	MLH1/PMS2 MSH2/MSH6	dMMR
4	BAT-26, NR-21, NR-24	High	PMS2	dMMR
5	MONO-27, NR-24	High	MSH2/PMS2	dMMR
6	BAT-26, NR-21, BAT-25, NR-24	High	MLH1/PMS2 MSH2	dMMR
7	BAT-26, NR-21, BAT-25, NR-24	High	MLH1/PMS2	dMMR
8	BAT-26, NR-21, MONO-27	High	MLH1/PMS2	dMMR
9	BAT-26, NR-21, BAT-25, MONO-27, NR-24	High	MLH1/PMS2	dMMR
10	NR-21, BAT-25	High	MSH2/MSH6 PMS2	dMMR
11	BAT-26, NR-21, BAT-25, MONO-27, NR-24	High	MLH1/PMS2	dMMR
12	BAT-26, NR-21, BAT-25, MONO-27, NR-24	High	PMS2	dMMR
13	BAT-26, NR-24	High	None	pMMR
14	NR-24	Low	None	pMMR
15	NR-24	Low	None	pMMR
16	BAT-25	Low	None	pMMR

### Concordance between IHC and MSI analysis

Of the 50 cases analysed for MSI, there are 2 cases with discordant results between MSI analysis and IHC. Of the 2 discordant cases, one case was detected as MSI-H by MSI analysis but pMMR by IHC and one case was identified MSS by MSI analysis but dMMR by IHC. There was perfect agreement between MSI analysis and IHC method with kappa value = 0.896 and p ˂ 0.001. The sensitivity and specificity for MSI analysis was 97.3% and 92.3%, respectively. The details of the cases exhibiting agreement between the two methods are shown in Table 5.

**Table 5:** Agreement between MSI analysis and MMR-IHC assay done on the CRC cases (*n* = 50)

	MSS/MSI-Low (*n*)	MSI-High (*n*)	Total	Kappa consistency test
pMMR	36	1	37	0.896 p˂0.001
dMMR	1	12	13	
Total	37	13	50	

pMMR: proficient Mismatch Repair; dMMR: deficient Mismatch Repair; MSS: Microsatellite Stable; MSI: Microsatellite instability; n: number of cases; p is significant at value ˂ 0.05

## Discussion

Assessment of all CRC patients for MMR/MSI status is a routine practice in clinical settings considering the effectiveness of newer immune checkpoint inhibitor treatment for advanced-stage dMMR/MSI-H CRC and underlying LS [15, 29–31]. In our study, we found 26% (13 out of 50) of the CRC cases were MSI-H, 6% (3 out of 50) cases were MSI-L and 68% (34 out of 50) cases were MSS. Studies in Malaysia have reported 15.1% (11 out of 73) [26] and 23.3% (10 out of 43) [32] CRC cases as MSI-H. However, the frequency ranged from 6.50% [33] to 36.1% (35 out of 97) MSI-H cases [19] in Chinese population. There is selection bias of cases in our study since 26% (13 out of 50) of our cases were dMMR phenotype detected by IHC. This difference in the frequencies might be due to certain factors such as tumour staging, selection bias, utilization of various MSI panels, genetic predisposition, ethnic variation, environmental factors, and sample size [34].

The frequency of dMMR/MSI-H tumours also differs among different ethnic groups. McCabe et al. [35] investigated the frequency of dMMR/MSI-H tumours in black South African among other races. The observed results were 15% (22 out of 148) and 8% (9 out of 119) in black and other ethnic groups of South Africa, respectively, which demonstrated a higher percentage of dMMR/MSI-H tumours in Black South Africans. Malaysia with a multi-ethnic distribution where Malays, Chinese, and Indians are the three main ethnic communities among others. We evaluated the MMR/MSI status of cases concerning the races if any racial feature is crucial for the CRC pathogenesis. We did not notice any statistically significant difference between MSI status and ethnic groups (*p* = 0.629). The results of our study were strengthened by the findings reported in earlier publications [32, 36].

The 3 MSI-L cases in our study sample exhibited pMMR status resembling the previous findings [37]. This supports the usual consensus that MSI-L CRC tumours should be classified with MSS tumours rather than MSI-H [12]. There was no abnormal MSH6 immunostaining pattern in MSI-L cases which would have supported the possibility of MSH6 genetic mutation [38]. Furthermore, previous studies utilizing the original Bethesda panel have reported higher frequency of MSI-L status in early CRC [39]. By comparison, using the mononucleotide panel in the present study, the frequency of MSI-L was 6% (3 out of 50) which is comparable to an earlier study. This demonstrates that MSI-L status is not frequent finding in CRC which might be of clinical importance [37].

Staining heterogeneity or discordance of staining has been reported previously in colorectal cancer, in which tumors exhibit diffuse and strong staining admixed with no staining or tumors showing faint staining patterns [40, 41]. This phenomenon has also been reported with automated IHC instruments previously where the results of IHC could be interpreted by less experienced pathologists as false-positive or false-negative; in which case, re-testing is recommended [42]. The evaluation of pre-analytical and IHC method standardization has been recommended in cases of heterogeneous staining to ensure the fidelity of IHC procedure. Furthermore, heterogeneous staining should not be considered as pMMR or artifactual automatically [22] with the possibility of discrepant results in the assessment of weak staining based on the experience of the pathologist [43].

In our study, the results of IHC reporting were altered with re-interpretation as 7 of the dMMR cases were reclassified as pMMR. The discordant results of the IHC method noted in our cohort and reported previously [21] were due to the heterogeneous staining and scattered absent/weak staining. We suggest that cases exhibiting heterogeneous staining or weak staining should be re-assessed to avoid misdiagnosis as reported previously [23] and further agree with the suggestion that rather be investigated using molecular methods and potential sequencing [22] since this phenomenon is noted to show differences in MMR status that are essential for precise classification [41]. However, not all answers are gained to reply the questions, more efforts are needed to find out exact mechanisms behind the heterogeneous staining patterns.

The most sensitive mononucleotide marker in our study was NR-24 demonstrating instability in 24% (12 out of 50) in MSI tumours which is in line with findings of a study in Iran [44]. Several other studies reported BAT-26 the most sensitive unstable mononucleotide marker in Malaysian [26] and various Asian populations [17, 26, 45, 46]. In the current research, BAT-26 and NR-21 were the second most unstable markers exhibiting instability in 22% (11 out of 50). Furthermore, the least sensitive mononucleotide unstable marker in our study was MONO-27 exhibiting instability in 14% (7 out of 50) of MSI cases which were supported by a study in Iran [46]. The variation in the findings suggests that the molecular phenotype of unstable mononucleotide markers is not the same among various populations. The reasons could be racial differences in the incidence of the markers and differences in LS CRC compared to sporadic CRC [46]. Moreover, it would be appropriate to detect the most frequent markers in a population to represent a panel with fewer markers for the identification of MSI status in CRC patients. If the panel with fewer markers could not detect the abnormality, then other markers may be used later [47].

### Analysis of concordance between MSI analysis and IHC

Although up to 100% concordance rate has been obtained between the MSI analysis and IHC methods in previous studies [48, 49], there has been the observation of variable discordant results as well. In the current study, 4% (2 out of 50) of the CRC cases demonstrated discordant results. Of the 2 discordant cases, one was detected as MSI-H by MSI analysis but pMMR by IHC and one case that was detected as dMMR by IHC but MSS by MSI analysis. The results are in line with a recent study which obtained 3.1% discordance [19] and even higher rates of discordance with up to 10% are reported previously [50, 51]. Surprisingly, Lin et al. [52] has reported 32.9% discordance using the National Cancer Institute (NCI) panel in MSI-H CRCs.

This research obtained perfect agreement (0.896, Kappa test) between MSI analysis and IHC tests. The results are comparable with the findings of an earlier study noticing a kappa value of 0.956 [34]. In contrast, substantial and moderate agreement were obtained with kappa values of 0.769 [17] and 0.547 [16], respectively. Hence, discordance between the two techniques may justify that: (i) the findings of the MSI analysis demonstrated that some of the mutated genes lead to altering microsatellite status but without undermining the antigenic quality of the protein which causes false-negative results on IHC, (ii) Loss of expression of individual MMR proteins may not be satisfactory evidence to the existence of MSI-H cases keeping in mind the functional redundancy of MMR proteins. (iii) time of fixation, staining process and types of antibody may affect the result of IHC, (iv) there may be other MMR genes other than 4 MMR genes (MLH1, MSH2, MSH6, and PMS2) which could lead to false-negative result [33].

The use of IHC or MSI analysis as a primary screening tool for CRC patients is controversial. Each has advantages and disadvantages but none of them is perfect and absolute. American Society of Clinical Oncology (ASCO) and the National Comprehensive Cancer Network (NCCN) guidelines recommended both methods to be utilized for the assessment of MMR/MSI status in CRC patients [9, 53]. In China, the two tests were compared with a conclusion that MSI analysis is more accurate while IHC is much easier and cheaper, thus both are applicable in the clinical settings [33]. Additionally, consensus diagnosis was not achieved in almost 18% tumours and staining pattern was misleading/equivocal in nearly 13% of MSI-H CRC cases [54].

IHC is a routine method to assess MMR status in histopathology laboratories. It can be performed in FFPE samples, easily applicable, affordable, and capable to guide specific gene mutation screening. However, the reporting of IHC heavily rely on the analysis of the pathologists and tissue preparation techniques, and it can only determine a few MMR proteins [12].

The MSI analysis has many benefits: it is appropriate for FFPE tissue samples, it does not require experienced pathologist, it can detect MSI cancers that have non-functional MMR system but retained MMR proteins expression on IHC due to non-truncated missense mutation, it can recognize MMR abnormality due to mutations of MMR proteins that are not listed in the IHC panel [25], and it is comparatively less exposed to alteration than IHC following the administration of radiation and neoadjuvant therapy. On the contrary, tissue fixation can affect PCR reaction, it requires a well-equipped molecular genetic lab and expert staff, and it is comparatively more costly than the IHC method [49, 55]. A recent study suggested precise cellularity determination before the conduction of MSI analysis since it can lead to inaccurate results [45].

### Limitations of the study

MSI analysis was performed only on selected CRC cases thus the high prevalence of MSI-H status (26%) reported here was not representing the actual figure. However, the chosen cases were appropriate to show the concordance between the IHC and MSI methods of analysis. The molecular method employed is insufficient to dictate either somatic or germline mutation as the cause of the MSI or to specify the actual underlying mutation, as this will require different approaches to molecular research.

## Conclusion

This study demonstrated a perfect agreement between IHC and MSI analysis methods. Our findings suggest that the selection of the method of choice for the recognition of MMR/MSI status in CRC patients should be based on the presence of expertise in the area and availability of the required equipment. Both factors determine the feasibility of the method. Since IHC is an affordable, readily available, and reproducible method in most histopathological laboratories, we suggest that IHC may be used as a primary screening test for the determination of MMR/MSI status in CRC patients.

### Recommendations

Several guidelines have recommended universal screening of MMR/MSI status in CRC patients. The relative prevalence of dMMR/MSI-H status in our cohort of CRC cases supports the recent experts’ recommendation of testing MSI or MMR status on all cases of CRC.

## Data Availability

No datasets were generated or analysed during the current study.

## Abbreviations

CRC: Colorectal Cancer
MMR: Mismatch Repair
MSI: Microsatellite Instability
IHC: Immunohistochemistry
MSS: Microsatellite Stable
MSI-H: MSI-High
MSI-L: MSI-Low
dMMR: Deficient Mismatch Repair
pMMR: Proficient Mismatch Repair
LS: Lynch syndrome
NCI: National Cancer Institute
